# Time Allocation in Clinical Training (TACT): national study reveals Resident Doctors spend four hours on admin for every hour with patients

**DOI:** 10.1093/qjmed/hcaf141

**Published:** 2025-06-23

**Authors:** Sammy Arab, Karanjot Chhatwal, Thomas Hargreaves, Michela Sorbini, Sara El-Toukhy, Chiara J Vedi, Yusuf Alghabra, Jude Merzah, Stuart D Rosen

**Affiliations:** Imperial College School of Medicine, Imperial College London, London, UK; Department of Cardiovascular Medicine, Mayo Clinic, Rochester, USA; Imperial College School of Medicine, Imperial College London, London, UK; National Heart and Lung Institute, Imperial College London, London, UK; Barts and The London School of Medicine and Dentistry, Queen Mary University of London, London, UK; St Mary's Hospital, Imperial College Healthcare NHS Trust, London, UK; Nottingham Medical School, University of Nottingham, Nottingham, UK; Ealing Hospital, London Northwest University Healthcare NHS Trust, London, UK; Faculty of Life Sciences & Medicine, King's College London, London, UK; Colchester General Hospital, East Suffolk & North Essex NHS Foundation Trust, Essex, UK; National Heart and Lung Institute, Imperial College London, London, UK; Ealing Hospital, London Northwest University Healthcare NHS Trust, London, UK

## Abstract

**Background:**

Resident Doctors (RDs) in the UK must balance clinical training with non-clinical administrative tasks. Concerns increase over how these tasks impact their professional development, job satisfaction and patient care. The shift toward consultant-delivered care has led to a redistribution of responsibilities, increasing administrative burden on RDs.

**Aims:**

This study aims to quantify how RDs allocate time between patient-facing and non-patient-facing tasks and assess impact on job satisfaction.

**Design:**

National multicentre observational cohort study.

**Methodology:**

The Time Allocation in Clinical Training study is a multicentre, observational cohort study conducted over seven months (January–July 2024) at secondary NHS centres. 137 RDs, from Foundation Year 1 to Specialty Training Year 8, were observed for 4-hour periods, with time tracked using stopwatches for five task categories. An optional survey assessed job satisfaction, rated on a five-point Likert scale.

**Results:**

RDs spent 73.0% of their time on non-patient-facing tasks, and 17.9% on patient-facing activities. Women spent more time on non-patient-facing tasks compared to men (75.0% vs. 69.9%, *P* = 0.03). Junior RDs (Foundation years and Core trainees) spent significantly less time on patient-facing tasks compared to senior RDs (ST6-8) (38.4% vs. 17.8%; *P* = 0.004). Most RDs (62%) reported dissatisfaction with the administrative burden. Electronic health record users spent significantly more time on administrative tasks than paper-based record users (44.1% vs. 37.3%; *P* = 0.02).

**Conclusions:**

The study reveals a significant imbalance between clinical and administrative tasks, with excessive administrative workload contributing to RD dissatisfaction. Streamlining administrative duties and integrating digital solutions is crucial for improving job satisfaction, clinical development and healthcare delivery in the NHS.

## Introduction

In the United Kingdom (UK), medical graduates undergo postgraduate training as Resident Doctors (RDs) within the National Health Service (NHS).^1^ Approximately 50% of NHS doctors are trainees, regulated by the General Medical Council (GMC), and are required to demonstrate clinical competency to progress in their training.^2^^,^^3^ Adherence to these standards of knowledge, skills, and performance is essential to ensuring both patient safety and the delivery of high-quality care.^3^ However, the balance between providing services and fostering the clinical development of trainees is increasingly challenged by inefficiencies, particularly the high administrative burden faced by RDs.^3^^,^^4^

Over the last decade, the shift towards the ‘Consultant Delivered Care’ (CDC) model has become a prominent feature of the NHS workforce structure.^5^ Under this model, post-Completion of Clinical Training doctors take on greater responsibility for patient care, while RDs assume a larger share of non-clinical tasks.^6^ This redistribution of responsibilities aims to optimize resource use within the multidisciplinary team (MDT) by leveraging the greater clinical and leadership experience of consultant doctors.^6^ However, while CDC may improve resource efficiency, it does not always correlate with improved patient care outcomes. In fact, excessive non-clinical duties for RDs can result in poorer health outcomes and diminished patient satisfaction.^7^^,^^8^ Non-clinical tasks are a major contributor to job dissatisfaction among healthcare professionals^8^ and with the NHS facing a workforce retention crisis, addressing job satisfaction is critical. Given that RDs have the highest rates of attrition within the NHS, this issue is even more pressing.^9^

Previous interventions to address workplace challenges for RDs have focused on regulating working hours.^10^ However, no evidence-based guidelines exist for the optimal balance between patient-facing and non-patient-facing tasks.^11^^,^^12^ The Time Allocation in Clinical Training (TACT) study addresses this gap by quantifying how RDs spend their time. Our findings highlight the need to optimize this balance to improve both doctor training and patient care outcomes.

Numerous NHS reform proposals have been made^13^^,^^14^ to improve efficiency and optimize resource allocation within the NHS, such as new digitalisation strategies^15^^,^^16^ and the introduction of new allied healthcare roles, such as Physician Associates (PAs) aimed to ease staff shortages and support RDs.^17^ However, it remains unclear whether these changes have had the desired impact on the clinical training experience of RDs, particularly in terms of reducing their administrative burden. The TACT study also evaluated trainee satisfaction to identify key challenges faced by RDs and highlight opportunities for improvement in healthcare delivery and medical education.

## Materials and methods

The TACT study is a national, multicentre observational cohort study designed to evaluate the distribution of NHS doctors’ time between patient-facing and non-patient-facing tasks and its impact on their clinical development. A secondary objective is to assess how this time allocation affects job satisfaction. Data collection was conducted over a 7-month period, from 7 January 2024 to 5 July 2024.

### Participants

RDs employed in secondary and tertiary NHS centres were recruited for the study. Clinical-year medical students were recruited as independent observers to track and record the activities of study participants.

**Inclusion Criteria:**

- RDs working in secondary care, enrolled in any GMC-approved postgraduate training programme.
- RDs in training grades ranging from Foundation-Year 1 (FY1) to Specialty-Training 8 (ST8).

**Exclusion Criteria:**

- RDs working in primary care or non-training posts.

### Stopwatch data collection

Medical student observers were trained in the use of a tool consisting of multiple stopwatches to track the time RDs spent on different work tasks. Five distinct stopwatches were used to categorize tasks as patient-facing or non-patient-facing and further sub-categorized into doctor-specific and non-doctor-specific tasks.

**Patient-Facing Task Tracking:**

- **Stopwatch 1** (Doctor-specific tasks): History taking, physical examinations, counselling.
- **Stopwatch 2** (Non-doctor-specific tasks): Procedural tasks such as cannulation, venepuncture, arterial blood gas sampling, surgery.

**Non-Patient-Facing Task Tracking:**

- **Stopwatch 3** (Doctor-specific tasks): Discussions with MDT, referrals, prescribing, interpretation of investigations.
- **Stopwatch 4** (Non-doctor-specific tasks): Administrative tasks including documentation, discharge summaries; chasing investigation results.
- **Stopwatch 5**: Other tasks; attending teaching sessions, taking work breaks.

Observers recorded task durations during a continuous 4-hours observation period, with each stopwatch measuring the time spent in minutes by RDs on corresponding activities.

### Satisfaction survey

Following the observation period, RDs were invited to complete an optional survey to assess their job satisfaction. Satisfaction was rated on a five-point Likert scale, with higher scores indicating greater satisfaction.

### Statistical analysis

Raw data from the stopwatch recordings and satisfaction surveys were collected via Google Forms and Qualtrics, respectively. The data were subsequently analysed using RStudio software (version 4.3.1, University of Auckland, New Zealand).

The Kolmogorov-Smirnov test was used to assess the distribution of the outcome variables: time spent on each stopwatch task and satisfaction ratings. Stopwatch data are presented as a percentage of the total observation period (in min).

Data were stratified by trainee grade, type of documentation system (electronic vs. paper-based), and observation duration. Pairwise comparisons were made using the Mann–Whitney U test throughout. Descriptive statistics for time spent within stopwatches are reported as median values ± interquartile range. A ranking plot was used to visualize work task preferences. Statistical significance was defined as *P* ≤ 0.05.

## Results

### Demographic data

A total of 137 observations were recorded, with a median observation duration of 240.0 (IQR 11.3) minutes. Data collection was distributed across morning (62%) and afternoon (38%) sessions. Of the observed RDs, 86 (62.8%) were foundation-doctors, while 51 (37.2%) were CT1/ST1-ST8. The mean age of participants was 28.0 years (SD 4.44). Detailed characteristics of the doctors’ demographics are summarized in Table 1.

**Table 1:** Detailed characteristics of the observed doctors’ demographics

	*N*	Male	Female	*P
**Total**	137	50	84	
**Ethnicity**				
White	47	19	28	
Non-white	84	31	53	0.71
**Grade**				
FY1-2	86	35	48	
CT/ST1-ST8	51	15	36	0.15
**Speciality**				
Medicine	92	38	54	
Surgery	15	5	7	
Acute Care	13	4	9	
Other	17	3	14	0.28
**Time**				
Morning	85	32	51	
Afternoon	52	18	33	0.72
**Note Type**				
Electronic	94	29	62	
Paper	43	21	22	0.08

* *P*-values calculated using Fisher’s exact test.

### Overall distribution of task durations

**Figure 1.**
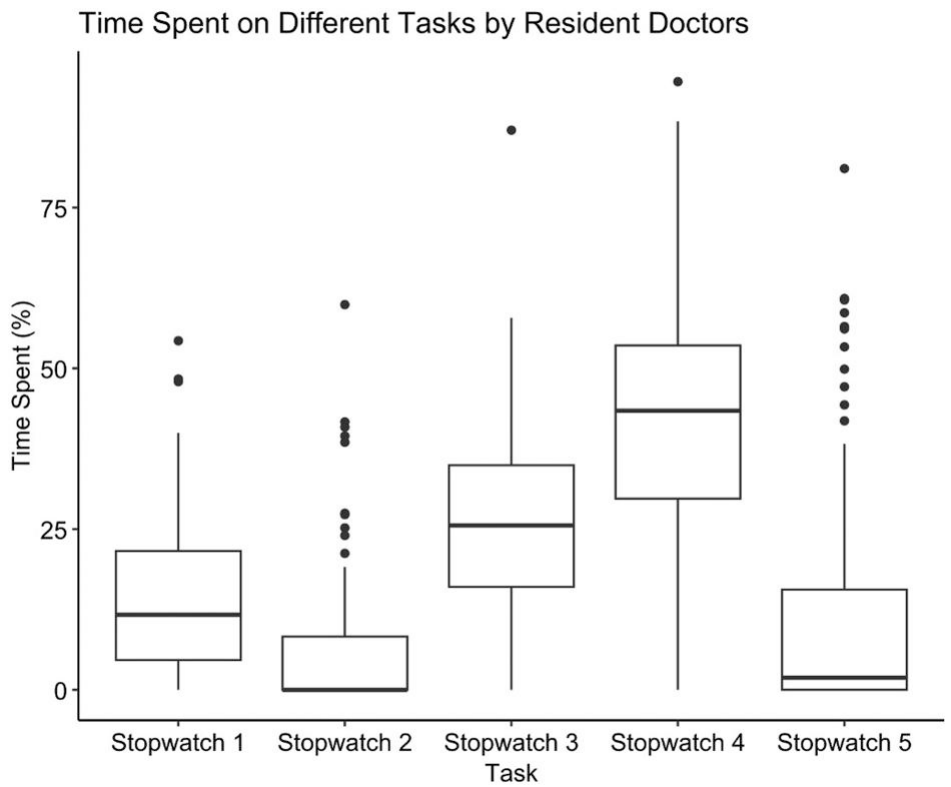
Box plot representing the distribution of time spent performing each task type. Time is represented as a percentage of the 4-hours observation period median (IQR). Non-patient-facing tasks occupied the greatest proportion of residents’ time.

The median time spent on patient-facing tasks, as recorded by Stopwatch 1 and Stopwatch 2, was 17.9% (IQR = 19.0) of the total observation time (Figure 1). In contrast, non-patient-facing tasks, as captured by stopwatches 3 and 4, occupied 73.0% (IQR = 24.1) of the observed time. Stopwatch 5 was used to capture tasks not covered by predefined categories, such as teaching sessions (90.6%) and breaks (28.0); in this stopwatch, some of the observations reported multiple activities. Overall, 10.8% (IQR = 16.5%) of the observation period was devoted to stopwatch 5 tasks, with a median of 1.89% (IQR = 15.6). Doctors spent ∼4 times more time on non-patient-facing administrative duties (Stopwatch 4) compared to doctor-specific, patient-facing tasks (Stopwatch 1) (43.4% vs. 11.7%, *P* < 0.001).

### Time allocation by demographic

**Figure 2.**
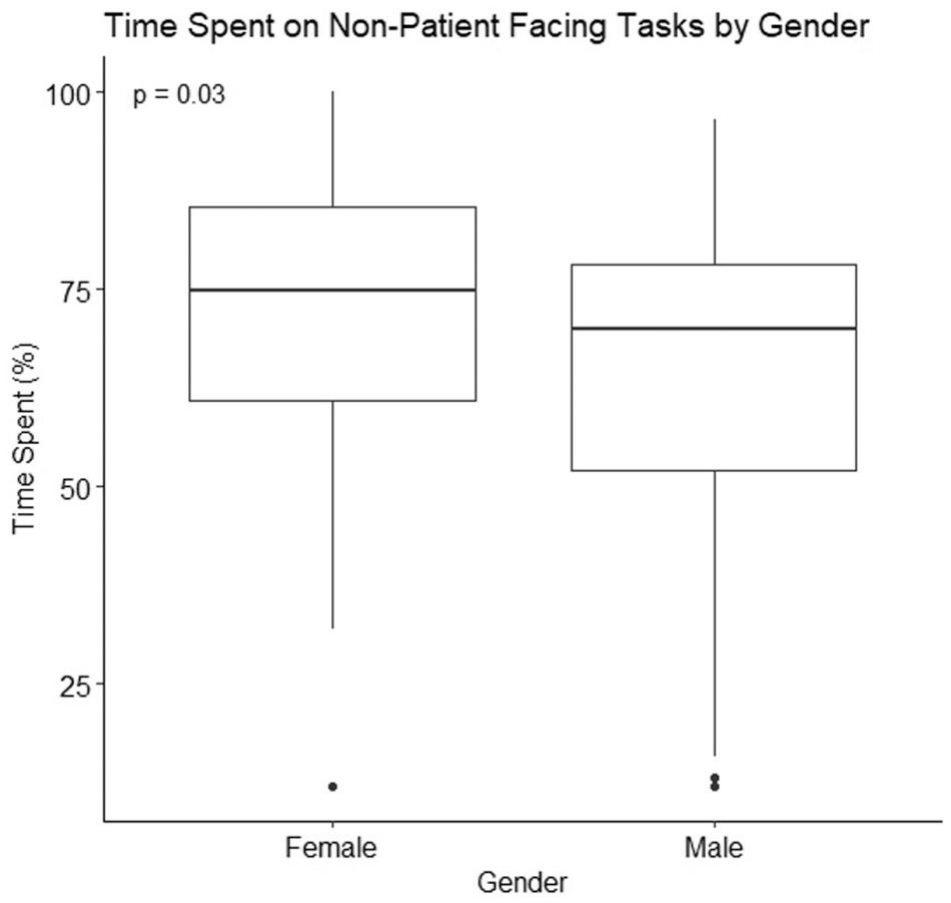
Gender differences in time spent on non-patient-facing tasks. The figure illustrates the percentage of time spent on non-patient-facing tasks (Stopwatch 3 and 4) compared to patient-facing tasks (Stopwatch 1 and 2) between male and female doctors. Women spent significantly more time on non-patient-facing tasks than their male counterparts (*P* = 0.03), highlighting a gender-based disparity in task allocation. Data are presented as median (IQR).

Analysis by gender revealed that female doctors spent a significantly higher proportion of their time on non-patient facing tasks compared to their male counterparts (75.0% vs. 69.9%, *P* = 0.03) (Figure 2). There were no significant differences in proportion of time spent on patient-facing activities across different ethnicities.

### Time allocation by training grade

**Figure 3.**
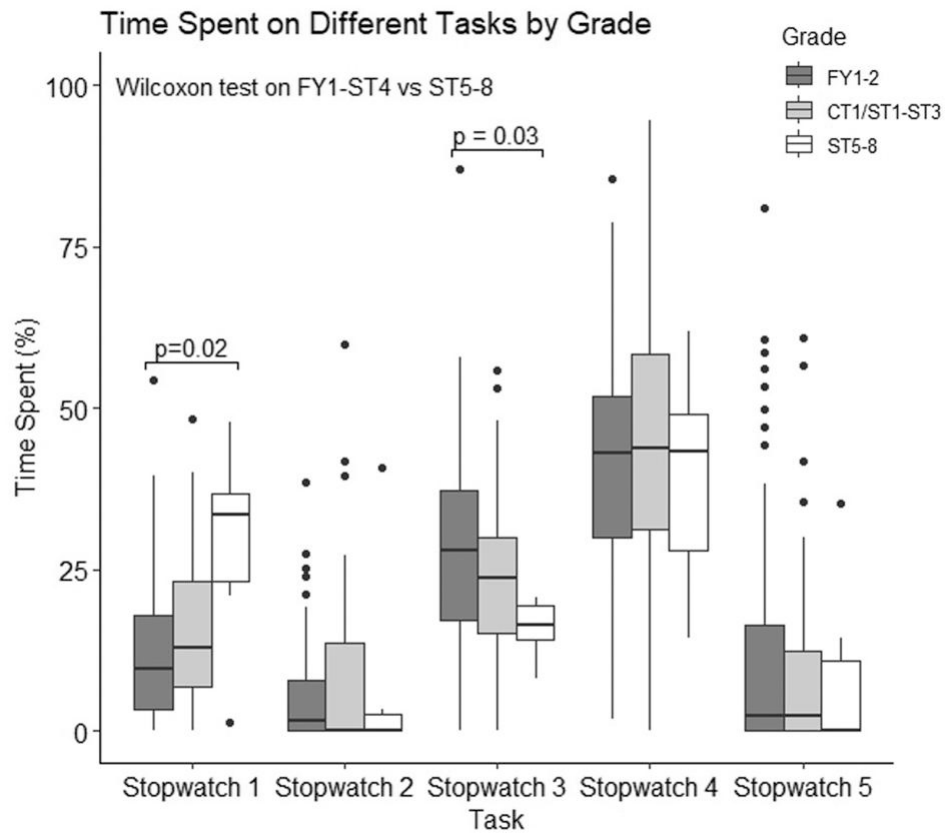
Box plot representing the distribution of time spent performing each task type, by different training grades. Time is represented as a percentage of the 4-hours observation period. Higher-grade trainees (≥ST3) were observed to spend the most time on patient-facing tasks.

Analysis by training grade revealed that senior doctors (ST6-8) spent a notably higher proportion of their time on patient-facing tasks compared to junior RDs (FY1-ST5). Specifically, ST6-8 doctors spent more time on patient-facing tasks (38.4% vs. 17.8%; *P* = 0.004), indicating a greater focus on direct patient care among more experienced doctors (Figure 3).

### Documentation systems

**Figure 4.**
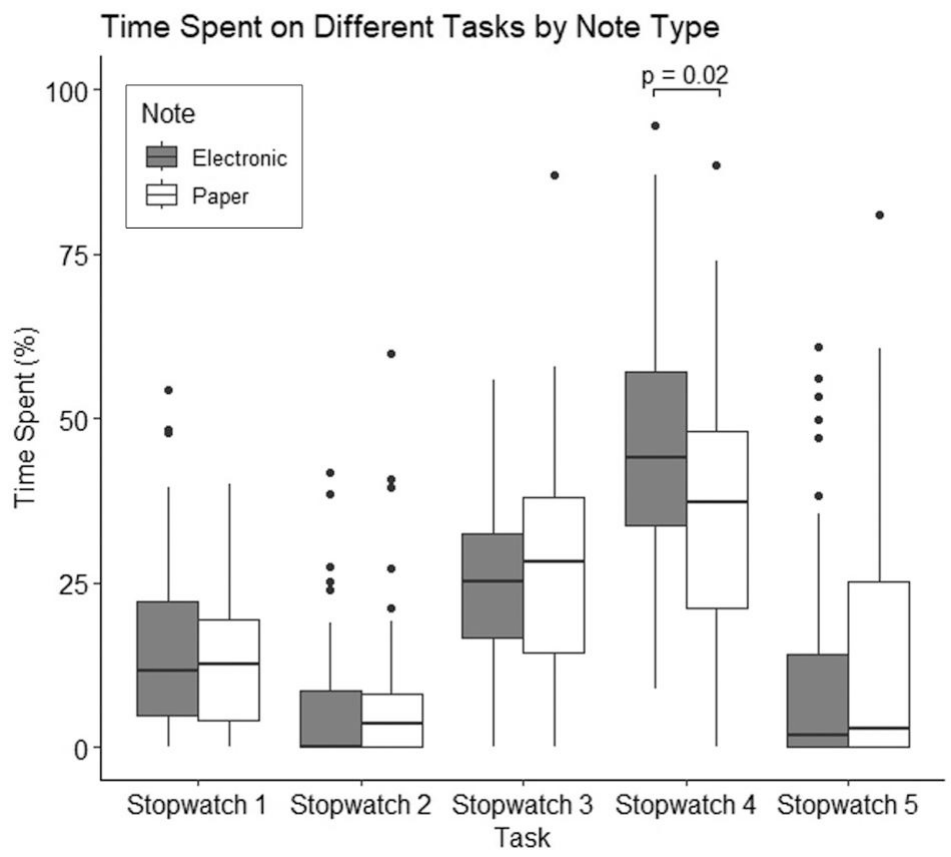
Box plot representing the distribution of time spent performing each task type, by documentation system. Time is represented as a percentage of the 4-hours observation period. Doctors using electronic records systems spent more time on Stopwatch 4: non-patient-facing non-doctor-specific tasks.

Electronic health records (EHRs) were used more often than paper-based notes (68.6% vs. 31.4%) (Figure 4). Doctors using paper systems spent significantly less time on Stopwatch 4 tasks (non-patient-facing, non-doctor-specific) than those using electronic systems (37.3% vs. 44.1%; *P* = 0.02). Among EHR users, 11 used Cerner and 18 used EPIC, with no significant difference in Stopwatch 4 time between the two systems.

### Observation timing

**Figure 5.**
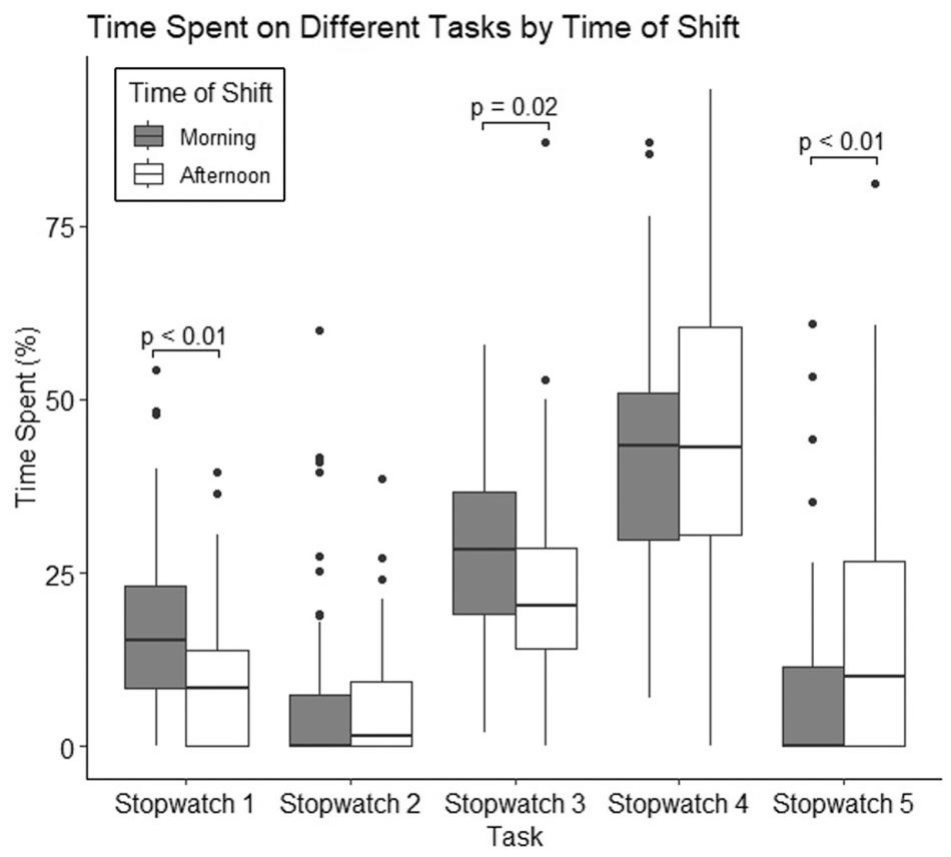
Box plot representing the distribution of time spent performing each task type, by timing of observation period. Time is represented as a percentage of the 4-hours observation period. Doctor-specific tasks, both patient-facing and non-patient-facing, predominated morning hours of shifts.

Time allocation also varied based on the time of day. Morning sessions showed significantly more time allocated to Stopwatch 1 (15.3% vs. 8.4%; *P* < 0.01) and Stopwatch 3 (28.3% vs. 20.3%, *P* = 0.02), while afternoon sessions had more time allocated to Stopwatch 5 tasks (0.0% vs. 10.1%, *P* < 0.01) (Figure 5).

### Satisfaction survey

**Figure 6.**
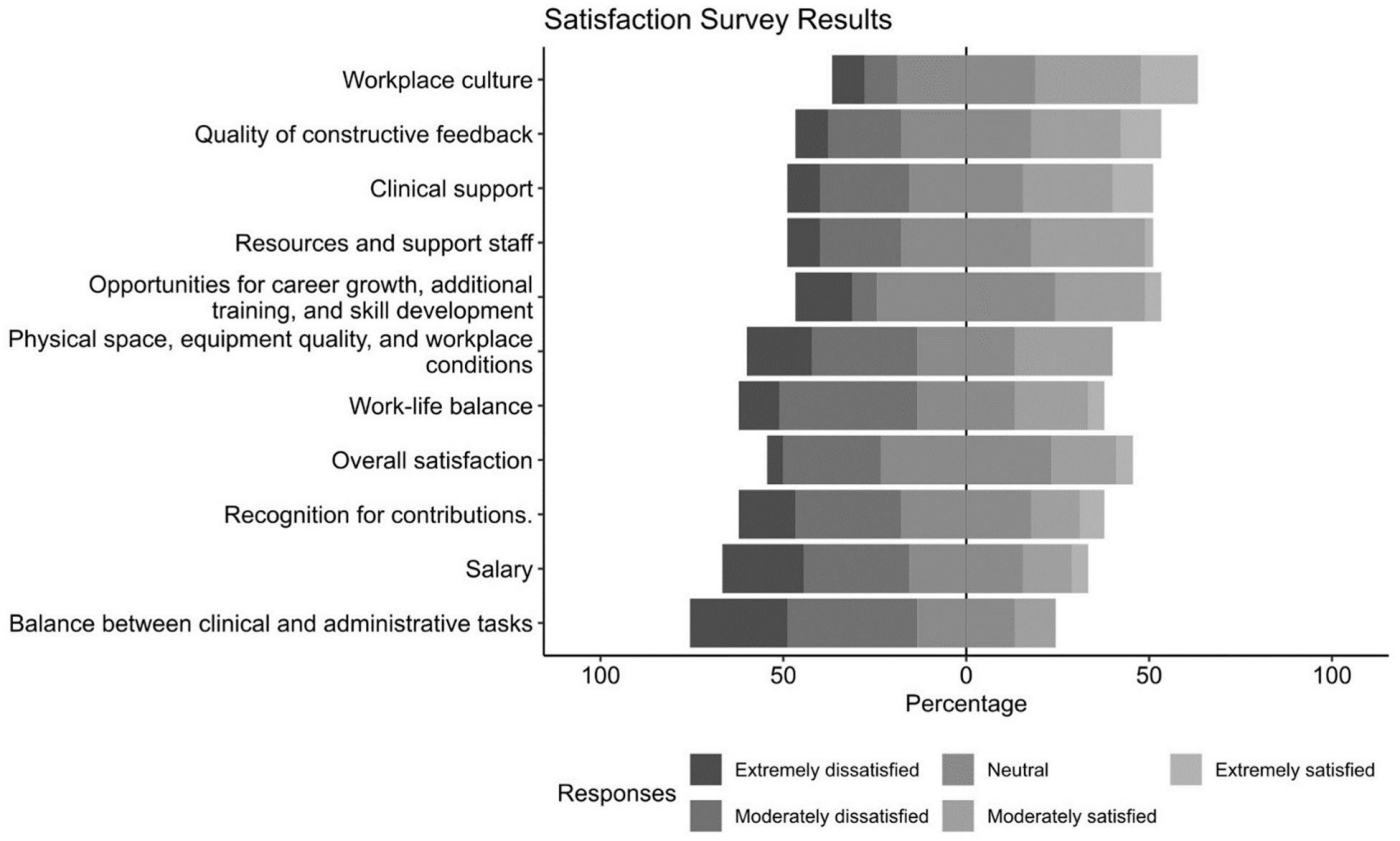
Five-point Likert scale results of RDs’ satisfaction with NHS training experience.

The satisfaction survey had a 32.8% response rate (45 respondents): 15 Foundation Doctors, 18 ST1/CT1–ST4 doctors, and 12 Registrars (ST5–8). Satisfaction was mixed: 10 reported being moderately or extremely satisfied, while 14 reported moderate or extreme dissatisfaction (Figure 6). Most dissatisfaction related to the balance between clinical and administrative tasks, with 28 expressing moderate or extreme dissatisfaction and only 5 were satisfied.

**Figure 7.**
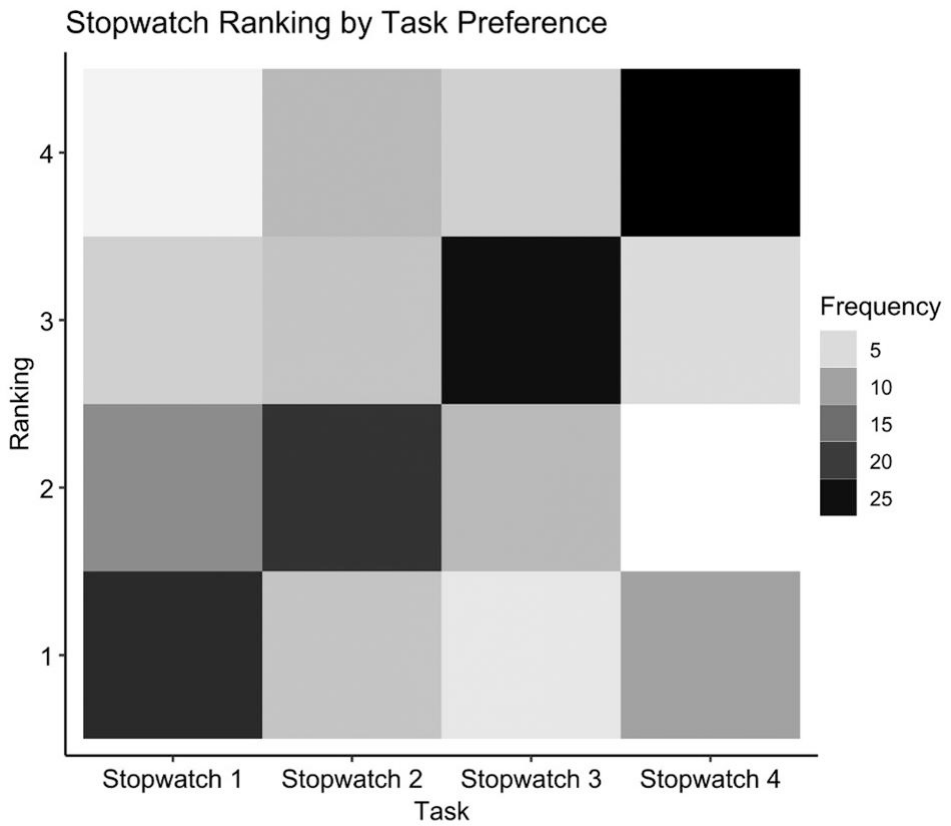
Rank plot showing RDs’ task preferences.

Additionally, 43 respondents ranked their work tasks from 1 (most preferred) to 4 (least preferred). Stopwatch 1 tasks were ranked first or second by 34 participants, Stopwatch 2 tasks by 28, and Stopwatches 3 and 4 by only 12 participants (Figure 7).

## Discussion

This national, multicentre, prospective observational study quantified the time allocation of RDs across various tasks, providing empirical evidence of their current workload distribution. We identified a significant imbalance between the time spent on administrative tasks (73.0%) versus direct patient care (17.9%), which contributes to dissatisfaction among RDs. In our survey, more doctors expressed dissatisfaction with administrative tasks (62.1%) than those who were dissatisfied with low pay (51.1%). While the recent pay restoration dispute led to strikes, our findings suggest that excessive administrative burdens may be a more fundamental driver of RD attrition, an issue that cannot be resolved through pay restoration alone. These findings align with the broader concerns around workforce retention and job satisfaction which are increasingly important in the current healthcare environment.

The GMC competencies^18^ serve as the framework for RD training programmes in the UK.^1^ However, the assessment of these competencies, which is primarily time-based through the Annual Review of Competency Progression, does not fully account for how RDs spend their time during training.^1^ Previous research has suggested an increasing administrative burden for RDs over the years, contrasting with data from the 1990s and early 2000s, when doctors spent 60–70% of their shifts on direct clinical care—a figure four times higher than what we observed in our study.^19^

One possible explanation is the shift towards a ‘consultant-delivered’ model of healthcare, where senior doctors take on more direct patient-facing roles, leaving RDs with more supporting tasks, such as documentation and administrative responsibilities.^5^^,^^6^^,^^20^ While this approach has been shown to improve efficiency, it contributes to trainee dissatisfaction, as our results confirm.^20^ Junior doctors are spending more time on administrative tasks, which may hinder their opportunities for clinical skill development.

Our study revealed that senior RDs (ST6-8) spent a higher proportion of their time on direct patient care (38.4% vs. 17.8%; *P* = 0.004) compared to junior RDs (FY1-ST5). This inter-grade disparity suggests that more experienced doctors have more opportunities for developing their communication skills, greater procedural proficiency, enhanced clinical judgment, and better adaptation to unpredictable clinical scenarios, while junior RDs may be missing out on crucial hands-on training. This finding aligns with our observation that only 17.9% of overall time was spent on patient-facing tasks, highlighting a potential gap in experiential learning for foundation-year doctors. Structured teaching rounds or supervised patient encounters may help address this gap and better balance service delivery with training needs.

While administrative tasks consumed 40% or more of RDs’ time across all training grades, not all such activities are devoid of educational value. For instance, engaging with clinical guidelines or summarizing patient care in discharge documentation may reinforce clinical reasoning. However, excessive administrative burden, particularly when repetitive or clerical in nature was consistently reported as a source of dissatisfaction and may detract from patient-facing experiential learning opportunities. Thoughtful redistribution of appropriate non-clinical tasks to trained allied healthcare professionals, such as PAs or ANPs could relieve some of this burden provided safeguards are in place to preserve documentation quality and continuity of care, especially in acute settings.

RD dissatisfaction in our study mirrors broader workforce trends. The 2023 GM workforce report shows RD attrition rates are double those of Consultants and GPs,^9^ with 62% of our respondents citing administrative burden as their main dissatisfaction, even exceeding concerns about salary. Since job satisfaction is crucial for retention, addressing this imbalance is key to curbing the NHS’s RD exodus.^21^^,^^22^

The knock-on effects of RD dissatisfaction and burnout have significant repercussions for patient care. Reduced patient contact can delay diagnoses, limit thorough assessments, and hinder communication among team members, increasing errors, and fragmented care. An overstretched workforce contributes to longer waiting lists, poorer outcomes, and declining trust in the healthcare system.^23^^,^^24^

Our results add to the debate on whether current training adequately prepares new doctors. While ‘student assistantships’, as recommended by the GMC, offer some preparation for clinical practice,^25–27^ our results suggest RDs still feel overwhelmed by administrative tasks. We propose integrating IT and AI training into the undergraduate curriculum to equip graduates with essential digital skills. Delegating non-clinical tasks such as drafting discharge summaries to allied healthcare professionals (AHPs), including PAs, could further reduce doctor workload. In Japan, this approach reduced paperwork by nearly 30%, alleviated fatigue and allowed doctors to focus more on complex clinical decision making.^25^^,^^26^ Expanding similar strategies in the NHS could relieve pressure on RDs and improve workforce efficiency.

Our study found that doctors using EHRs spent more time on administrative tasks than those using paper records, suggesting inefficiencies despite EHRs being designed to streamline workflows. AI and automation could address this, as seen with the DrDoctor platform, which reduced waiting times by automating appointment bookings.^28^ AI tools could also generate discharge summaries and automate other routine tasks, freeing doctors for clinical care. In primary care, an estimated 44% of administrative tasks are automatable.^29^ However, integrating AI requires safeguards for data security, and more critically, robust oversight to prevent errors from immature systems like generative AI, which may produce incorrect diagnoses or treatment recommendations.^30^

Our study suggests senior doctors engage more directly with patients; structured mentorship programs pairing them with junior trainees could help bridge gaps in clinical exposure, provided staffing levels are sufficient to balance education and care.^31^^,^^32^

Qualitative feedback from observers highlighted key inefficiencies in RD workflows, notably excessive time spent on tasks that could be automated or delegated, and fragmented patient records across NHS hospitals. Observers frequently reported RDs manually retrieving imaging and test results and writing discharge summaries, tasks better suited to ancillary staff or AHPs. Fragmented records further compounded delays, prompting calls for a unified national EHR system to streamline documentation. Observers also noted frequent computer system failures causing delays of up to 30 min, underscoring the urgent need for improved digital infrastructure to support efficient clinical workflows.

These findings should be interpreted in light of the study’s limitations. First, observer subjectivity may have affected time categorisation, especially during multitasking, leading to potential inter-rater variability despite standardized instructions and forms. We prioritized recording patient contact to maintain accuracy. Second, the satisfaction survey may be subject to reporting bias, as participants likely skewed toward dissatisfied RDs, limiting generalisability to the wider RD population. Third, the scope of our observations was limited to interactions involving RDs. As data collectors were medical students, we did not have the institutional access to observe allied healthcare professionals such as PAs. As such, we were unable to evaluate the role or impact of allied healthcare professionals within clinical teams. Fourth, while subgroup analysis by age and ethnicity was conducted and found no statistically significant differences, the study was not powered to detect such effects; future research should explore how demographic factors influence perceived task allocation and burden. Finally, as a cross-sectional study, our data represents a single timepoint and may not reflect temporal fluctuations in workload due to seasonal or policy changes. Longitudinal studies are needed to assess how workload and satisfaction evolve over time, particularly in response to interventions targeting administrative burden.

## Conclusion

This study provides strong evidence that the current workload distribution for RDs in the UK, dominated by administrative tasks, is harming professional development and job satisfaction. Addressing this imbalance requires urgent reforms, including task redistribution, integrated digital solutions, formal mentorship programs, improved IT infrastructure, AI task automation, and a unified EHR system. These measures are essential to enhance RD efficiency, job satisfaction, and patient care, while ensuring a sustainable, effective NHS and retaining a motivated medical workforce from undergraduate training onward.
